# Polyhydroquinone-graphene composite as new redox species for sensitive electrochemical detection of cytokeratins antigen 21-1

**DOI:** 10.1038/srep30623

**Published:** 2016-07-28

**Authors:** Huiqiang Wang, Qinfeng Rong, Zhanfang Ma

**Affiliations:** 1 Department of Chemistry, Capital Normal University, Beijing, 100048, China

## Abstract

Polyhydroquinone-graphene composite as a new redox species was synthesized simply by a microwave-assisted one-pot method through oxidative polymerization of hydroquinone by graphene oxide, which exhibited excellent electrochemical redox activity at 0.124 V and can remarkably promote electron transfer. The as-prepared composite was used as immunosensing substrate in a label-free electrochemical immunosensor for the detection of cytokeratins antigen 21-1, a kind of biomarker of lung cancer. The proposed immunosensor showed wide liner range from 10 pg mL^−1^ to 200 ng mL^−1^ with a detection limit 2.3 pg mL^−1^, and displayed a good stability and selectivity. In addition, this method has been used for the analysis of human serum sample, and the detection results showed good consistence with those of ELISA. The present substrate can be easily extended to other polymer-based nanocomposites.

Electrochemical immunesensors are widely used because of their simple preparation, fast detection, and high sensitivity^1^,^2^,^3^. Hence, electrochemical immunosensors have become one of the major analytical techniques for detecting biomolecules. Among these electrochemical immunoassays, label-free electrochemical immunoassay is attractive for its easy fabrication, low-cost and time-saving^4^,^5^,^6^,^7^,^8^,^9^,^10^. Commonly, the construction of label-free electrochemical immunosensor is mainly based on the sensing substrate nanomaterials with good conductivity, excellent stability and large specific surface area for antibodies attachment^11^,^12^.

At present, however, most of substrate materials do not possess redox property. In this case, redox species which are indispensible for label-free electrochemical immonosensors were usually used as following three ways. First, the reversible redox species were added in electrolyte solutions with high concentration, such as ferricyanide ([Fe(CN)_6_]^4−/3−^), ferrocene and dyes^13^,^14^,^15^. This way possibly results in lowering the bioactivity of antibodies or antigens. Second, the redox species was modified directly on the substrate materials by chemical bonds^16^,^17^,^18^. This will make the modification process of electrode complicated and cumbersome due to the multiple chemical coupling processes involved. Third, the redox species were fixed directly on electrode and then covered by polymer film^19^. However, there is the possibility of leakage for redox species. Given the above situations, it is very necessary to develop a new type of nanomaterial-based substrates with good conductivity and chemical stability, particularly with intrinsic redox activity, for label-free electrochemical immunosensor.

In this work, polyhydroquinone-graphene composite (rGO&PHQ) as a new redox species was synthesized simply by a microwave-assisted one-pot method through oxidative polymerization of hydroquinone by graphene oxide. This composite exhibited excellent electrochemical signal at 0.124 V and can remarkably promote electron transfer. rGO&PHQ as a substrate was used to fabricate a sensitive label-free amperometric immunosensor. Cytokeratins antigen 21-1 (CYFRA21-1) was chosen as a model analyte to exam the analytical performance of the proposed immunosensor. Superior performance for CYFRA21-1 detection with low detection limit, wide detection range, high sensitivity, and good stability was reached.

## Results and Discussion

**Figure 1:**
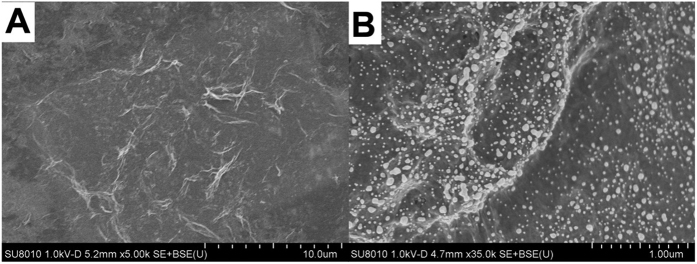
SEM images of rGO&PHQ (**A**) and rGO&PHQ-Au (**B**) composite.

The as-prepared rGO&PHQ and rGO&PHQ-Au composites have a lamellar structure, and gold nanoparticles uniformly distributed on rGO&PHQ-Au composite as shown in Fig. 1. The chemical compositions of rGO&PHQ and rGO&PHQ-Au composite were analyzed by energy dispersive X-ray spectroscopy (EDS). As shown in Figure S1, Si, C, O, and Si elements can be observed in rGO&PHQ. The Si element is displayed since silicon slice was used as the sample platform. Compared with rGO&PHQ, Au element in rGO&PHQ-Au composite can be observed. This indicated that the AuNPs are successfully deposited on rGO&PHQ composite.

**Figure 2:**
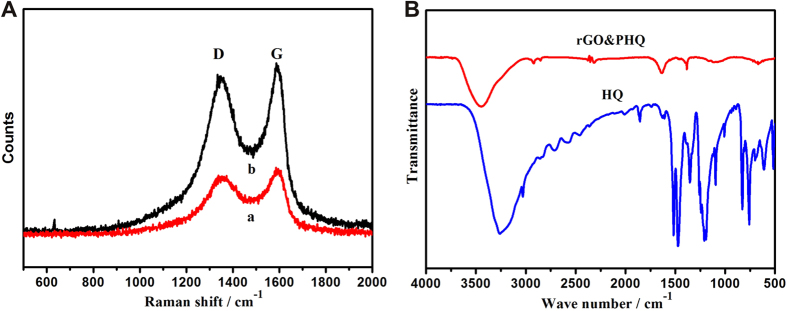
(**A**) The Raman spectra of GO (a) and rGO&PHQ (b). (**B**) The FT-IR spectrum of rGO&PHQ and HQ.

The Raman spectra of GO and rGO&PHQ composites were shown in Fig. 2. Two characteristics peaks are generally known D and G bands of GO. The D and G bands of GO were appeared at 1350 and 1597 cm^−1^, respectively, with the intensity ratio (I_D_/I_G_) of 0.99. In comparison with GO, the G band of rGO&PHQ composite slightly shifted to the lower wavenumbers which indicated the reduction in GO after the microwave irradiation^20^. The FT-IR spectrum of rGO&PHQ is quite different from that of HQ^21^. The broad band at 3500 cm^−1^ is attributed to the stretching of O-H in hydroxide or water. The strong band at 1651 cm^−1^ is assigned to the stretching of C=O. This band is stronger in the rGO&PHQ composite compared with that in HQ, because the phenolic hydroxyl groups in rGO&PHQ were oxidized by GO to be quinone structures. The bands at 1194 cm^−1^ and 756 cm^−1^ are associated with C-O stretching and C-H bending, respectively. During the experiment, the color of the sample changes from brown to dark, indicating the GO (brown) was reduced to be rGO (black).

**Figure 3:**
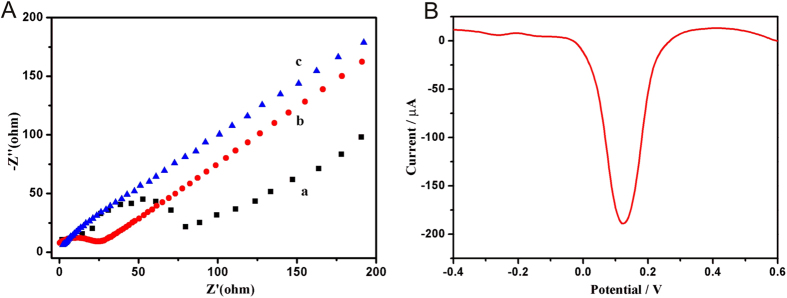
(**A**) The EIS of bare GCE (curve a), GCE modified with rGO&PHQ (curve b), and rGO&PHQ-Au (curve c). (**B**) The polyhydroquinone-graphene composite exhibited strong redox activity at 0.124 V.

Electrochemical impedance spectroscopy (EIS) was used to investigate the interfacial properties of the electrode modified with rGO&PHQ composite. The Nyquist plot of rGO&PHQ modified GCE (Fig. 3A, curve b) showed a semicircle with smaller diameter compared to the bare glassy carbon electrode (GCE) (Fig. 3A, curve a), which indicated a small charge transfer resistance. It was because the polyhydroquinone-graphene composite can remarkedly promote electron transfer ability. After the deposition of Au film, the resistance was further decreased (Fig. 3A, curve c), indicating the doposited Au film can effectively promote the electron transfer. Importantly, the polyhydroquinone-graphene composite exhibited excellent electrochemical redox activity at 0.124 V, which it is precondition to be used as immunosensing substrate in a label-free electrochemical immunosensor.

**Figure 4:**
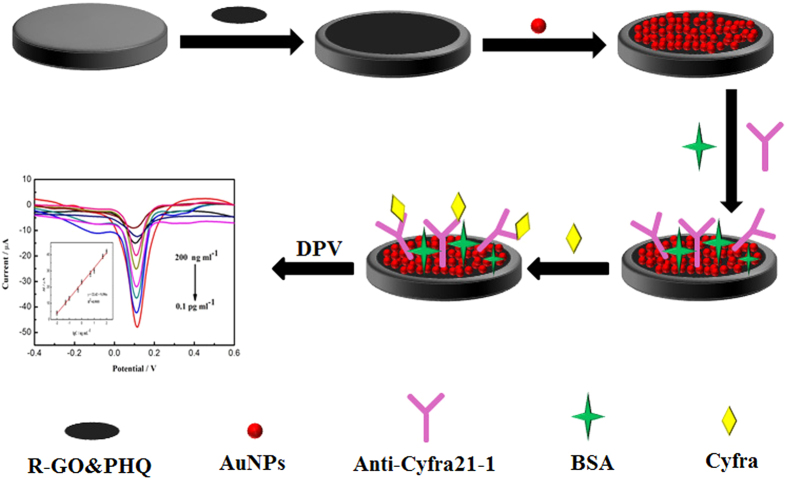
Schematic illustration of the fabrication process of the immunosensing interface.

For an electrochemical biosensor, its performance is critically dependent on the properties of the immunosensing interface^22^,^23^,^24^,^25^,^26^. The immunosensing substrate with reversible redox potential, high specific surface area, and excellent conductivity played a crucial role in the detection limit, linear range, and sensitivity^27^,^28^,^29^,^30^,^31^,^32^,^33^. The principle of the proposed immunosensor was illustrated in Fig. 4. A stable film of rGO&PHQ composite was easily formed on GCE simply by dropping method. Meanwhile, the electrodeposited AuNPs on the rGO&PHQ composite can further increase the specific surface area to capture a lot of antibodies as well as improve the capability of electron transfer. After fixing anti-CYFRA21-1 and blocking with BSA, the immunosensing interface was realized. Due to the poor conductivity of proteins such as CYFRA21-1 and anti-CYFRA21-1, the current decrease will be observed. The current responses at 0.124 V were proportionate to the logarithm values of CYFRA21-1 concentrations. In this case, the concentration of CYFRA21-1 can be measured.

**Figure 5:**
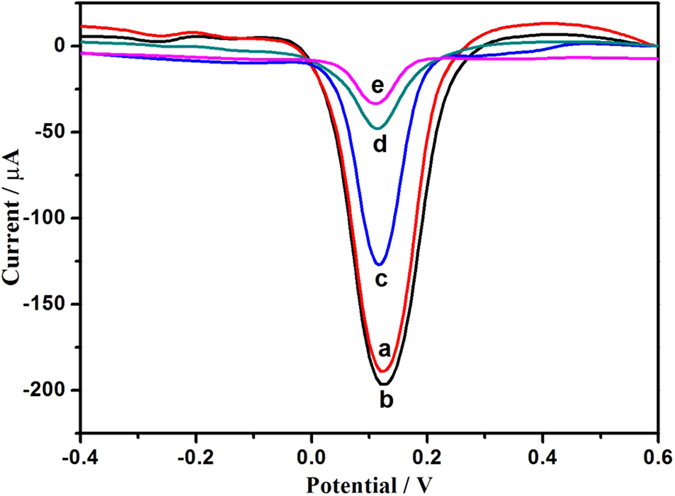
DPV responses of the modified procedure of electrodes in 0.1 M PBS-and 0.1 M KCl (pH 5.5) (a) rGO&PHQ composite modified GCE; (b) rGO&PHQ/Au composite modified GCE; (c) anti-CYFRA21-1/Au/rGO&PHQ composite modified GCE; (d) blocked with 1% BSA; (e) modified glassy carbon electrode after incubation with 1 ng mL^−1^ CYFRA21-1.

The fabrication process of the immunosensor was monitored by differential pulse voltammetry (DPV) in PBS buffer (Fig. 5). The current response of GCE modified with rGO&PHQ-Au composite (curve b) was higher than that of rGO&PHQ composite modified GCE (curve a). This was attributed to the excellent conductivity of AuNPs and the increase of the surface area of the electrode. In contrast, the loading of anti-CYFRA21-1 led to an obvious decrease of the peak current (curve c). The current response further decreased after the immunosensor was blocked with BSA (curve d) and incubated in a solution with 1 ng mL^−1^ CYFRA21-1 (curve e).

The pH value of detection solution directly influences the activity of the antibodies. In this case, the effect of the pH on the immune reaction was investigated. As shown in Figure S2A, with in increase of pH value, the peak current increased when the pH value was less than 5.5, and then decreased with the increase of pH value when pH value was more than 5.5. So, pH 5.5 was used in following experiments. In order to optimize the incubation time, the effect of the incubation time on the immune reaction was also studied. As shown in Figure S2B, the peak current increased with the increase of the incubation time, and then kept constant after 45 min. In this case, 45 min was used as the incubation time for the immunoassay.

**Figure 6:**
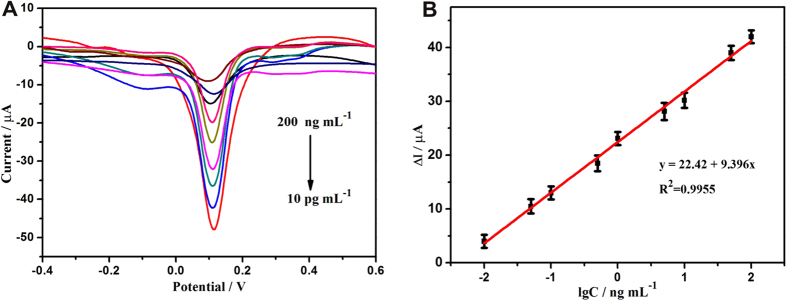
(**A**) DPV responses of electrochemical immunoassay in 0.1 M, pH 5.5 PBS, curves a–i correspond to CYFRA21-1 at the concentrations from 10 pg mL^−1^ to 200 ng mL^−1^. (**B**) The calibration plot between the DPV peak current and the logarithm values of CYFRA21-1 concentrations. Error bars represent standard deviation, n = 3.

The analytical performance of this immunosensor was tested by DPV. The current response of the immunosensor decreased with the increase of CYFRA21-1 concentration (Fig. 6A). The immunosensor displayed good linear relation ranging from 10 pg mL^−1^ to 200 ng mL^−1^ (Fig. 6B) with a low detection limit 2.3 pg mL^−1^ (the ratio of signal to noise (S/N) =3). In order to investigate the anti-interfering ability of the proposed immunosensor, the analytical performance using neuron-specific enolase (NSE), alpha fetoprotein (AFP), prostate specific antigen (PSA), UA, glucose, AA, BSA, and IgG as analyte instead of CYFRA21-1 was investigated. When the immunosensor was incubated with 100 ng mL^−1^ AA, UA, NSE, AFP, BSA, IgG, PSA, and glucose solution, respectively, no obvious changes of the current were observed compared to the blank test (no target molecule) in the same testing conditions as shown in Figure S3. However, when CYFRA21-1 was coexisted with the interferences, the electrochemical responses were almost the same as that with only CYFRA21-1. All these results indicated that the proposed immunosensor had a good specificity for CYFRA21-1. To further examine the stability of the immunoassay, the present immunosensor was stored at 4 °C for about four weeks and then its electrochemical property was measured. The change of current response was less than 10%, revealing that the stability of the immunoassay was good. The repeatability was also studied using three electrodes, and the relative standard deviation was 4.52%, showing that the repeatability of the immunosensor was acceptable.

In order to investigate the reliability and accuracy of the proposed immunosensor for clinical sample, sixteen clinical serum samples containing CYFRA21-1 were analyzed. The obtained results were compared with the ELISA tests (Table 1). It can be seen that the relative standard error was less than 6%, indicating this electrochemical immunoassay had a good accuracy for clinical sample detections. Thus, the immunosensor had a potential application for the detection of CYFRA21-1 in clinical diagnostics.

## Conclusion

In summary, a novel electrochemical redox-active polyhydroquinone-graphene composite was synthesized by a one-pot method by oxidative polymerization of hydroquinone by graphene oxide, which exhibited excellent electrochemical redox activity at 0.124 V and good conductivity. The as-prepared composite was used as sensing substrate in the fabricating label-free electrochemical immunosensor for the detection of cytokeratins antigen 21-1. The proposed immunosensor showed a good analytical performance. A wide detection ranges from 10 pg mL^−1^ to 200 ng mL^−1^ and the detection limit 2.3 pg mL^−1^ was reached. Besides, the detection results of human serum sample by this immunosensor were in good consistence with ELISA ones. This new sensing substrate can be easily extended to other polymer-based nanocomposites, which is of significance for the label-free electrochemical immunosensors.

## Methods

### Materials

Hydroquinone, graphene oxide and BSA were bought from Sigma-Aldrich. Ascorbic acid, hydrogen tetrachloroaurate hydrate (HAuCl_4_·xH_2_O, 99.9%), D-(+)-glucose, ascorbic acid, and uric acid were achieved from Alfa Aesar (Tianjin, Cina). CYFRA21-1, anti-CYFRA21-1, CEA, NSE, AFP and IgG were purchased from Shanghai Linc-Bio Science Co. Ltd. (Shanghai, China). Na_2_HPO_4_, NaH_2_PO_4_ and KCl were obtained from Beijing Chemical Reagents Company (Beijing, China). Clinical human serum samples were provided by Capital Normal University Hospital (Beijing, China). All the chemical reagents were of analytical grade and used without further purification.

### Apparatus

All electrochemical measurements were carried out on a CHI832 electrochemical workstation (Chenhua Instrumentsn Co., Shanghai, China). SEM images and EDS were determined with a Hitachi SU8010 SEM. Ultrapure water used in all procedures was purified through an Olst ultrapure K8 apparatus (Olst, Ltd., resistivity >18 MΩ). A three electrochemical system in the experiment was composed of a GCE (4 mm in diameter) as the working electrode, a Platinum wire and a Ag/AgCl electrode as counter electrode and reference electrode, respectively. Fourier transform infrared spectroscopy (FTIR) spectrum was obtained from FTIR spectroscopy (Tensor37, Bruker, Germany).

### Preparation of polyhydroquinone-graphene composite

The polyhydroquinone-graphene composite was synthesized by adding hydroquinone (1 mmol) to graphene oxide (0.5 mg mL^−1^, 5 mL), then microwave-assisted one-pot method through oxidative polymerization. The composites were centrifuged at 16000 rpm about 20 min and were washed with ultrapure water for several times. Ultimately, the composites were redispersed in 1 mL ultrapure water.

### Fabrication of immunosensor

The GCE was polished with alumina powders of 0.05 μm, which was followed by ultrasonic processing in ultrapure water and then dried by N_2_. After that, 15 μL solution of rGO&PHQ was dropped on the surface of a pretreated GCE and allowed for 30 min to form a thin homogeneous film. The electrode modified with rGO&PHQ was socked in HAuCl_4_ diluted solution, then a depositional potential of −0.2 V for 30 s was used to form Au film. Then the modified electrode was rinsed with ultrapure water. Then, 80 μL 200 μg mL^−1^ capture antibodies (anti-CYFRA21-1) were dropped on the rGO&PHQ/Au-modified GCE and incubated for 12 h at 4 °C to anchor the antibody to the surface of electrode. Subsequently, 20 μL 1 wt% BSA was used to block the nonspecific active sites. After washed with purified water, the modified electrode was obtained and stored in 4 °C before use.

## Additional Information

**How to cite this article**: Wang, H. *et al*. Polyhydroquinone-graphene composite as new redox species for sensitive electrochemical detection of cytokeratins antigen 21-1. *Sci. Rep*. **6**, 30623; doi: 10.1038/srep30623 (2016).

## Supplementary Material

Supplementary Information

## Figures and Tables

**Table 1:** Assay results of clinical serum samples using the proposed and ELISA.

Sample	Proposed immunosensor (ng mL^−1^)	ELISA (ng mL^−1^)	Relative error (%)
1	1.34	1.40	−4.28
2	1.21	1.18	2.54
3	1.48	1.50	−1.33
4	1.02	0.97	5.15
5	0.90	0.88	2.27
6	1.20	1.23	−2.44
7	0.90	0.85	5.88
8	0.50	0.48	4.16
9	0.52	0.54	−3.70
10	1.00	0.98	2.04
11	0.60	0.61	−1.64
12	1.00	0.99	1.01
13	0.60	0.58	3.45
14	0.68	0.69	−1.44
15	0.19	0.18	5.55
16	0.20	0.20	0.00

## References

[b1] Liu K. P., Zhang J. J., Wang C. M. & Zhu J. J. Graphene-assisted dual amplification strategy for the fabrication of sensitive amperometric immunosensor. Biosens. Bioelectron. 26, 3627–3632 (2011). 21388800 10.1016/j.bios.2011.02.018

[b2] Tang J. . Sensitive electrochemical immunoassay of carcinoembryonic antigen with signal dual-amplification using glucose oxidase and an artificial catalase. Anal. Chim. Acta 697, 16–22 (2011). 21641413 10.1016/j.aca.2011.04.022

[b3] Kong F. Y., Xu M. T., Xu J. J. & Chen H. Y. A novel lable-free electrochemical immunosensor for carcinoembryonic antigen based on gold nanoparticles-thionine-reduced graphene oxide nanoparticle film modified glassy carbon electrode. Talanta 85, 2620–2625 (2011). 21962692 10.1016/j.talanta.2011.08.028

[b4] Guo H. L., Wang X. F., Qian Q. Y., Wang F. B. & Xia X. H. A green approach to the synthesis of graphene nanosheets. ACS Nano 3, 2653–2659 (2009). 19691285 10.1021/nn900227d

[b5] Wang Z. J. . The synthesis of ionic-liquid-functionalized multiwalled carbon nanotubes decorated with highly dispersed Au nanoparticles and their use in oxygen reduction by electrocatalysis. Carbon 46, 1687–1692 (2008).

[b6] Xu W. J. . An electrochemical aptasensor for thrombin using synergetic catalysis of enzyme and porous Au@Pd core– shell nanostructures for signal amplification. Biosnes. Bioelectron. 64, 423–428 (2015). 10.1016/j.bios.2014.08.091 25280342

[b7] Zhang J., Yuan Y. L., Xie S. B., Chai Y. Q. & Yuan R., Amplified amperometric aptasensor for selective detection of protein using catalase-functional DNA–PtNPs dendrimer as a synergetic signal amplification label. Biosnes. Bioelectron. 60, 224–230 (2014). 10.1016/j.bios.2014.04.024 24813911

[b8] Fan H. X. . Ultrasensitive electrochemical immunosensor for carbohydrate antigen 72-4 based on dual signal amplification strategy of nanoporous gold and polyaniline-Au asymmetric multicomponent nanoparticles. Biosens. Bioelectron. 64, 51–54 (2015). 25194795 10.1016/j.bios.2014.08.043

[b9] Chen D. & Li J. H. Graphene oxide: preparation, functionalization and application in electrochemical sensors. Chem. Rev. 112, 6027–6053 (2012). 22889102 10.1021/cr300115g

[b10] Jia X. L., Chen X., Han J. M., Ma J. & Ma Z. F. Triple signal amplification using gold nanoparticles, bienzyme and platinum nanoparticles functionalized graphene as enhancers for simultaneous multiple electrochemical immunoassay. Biosen. Bioelectron. 53, 65–70, (2014). 10.1016/j.bios.2013.09.021 24113435

[b11] Yang L. J., Li Y. B. & Erf G. F. Interdigitated array microelectrode-based electrochemical impedance immunosensor for detection of Escherichia coli O157:H7. Anal. Chem. 76, 1107–1113 (2004). 14961745 10.1021/ac0352575

[b12] Huang H. Z., Ran P. X. & Liu Z. G. Impedance sensing of allergen-antibody interaction on glassy carbon electrode modified by gold electrodeposition. Bioelectrochemistry 70, 257–262 (2007). 17113360 10.1016/j.bioelechem.2006.10.002

[b13] Wang Y. . Gold nanoparticles-graphene nanosheets hybrid based biosensor: application to organophosphate pesticide and nerve agent detection. J. Mater. Chem. 21, 5319–5325 (2011).

[b14] Shao Y. Y. . Graphene based electrochemical sensors and biosensors: a review. Electroanal. 22, 1027–1036 (2010).

[b15] Zeng Q. . Self-assembled graphene-enzyme hierarchical nanostructures for electrochemical biosensing. Adv. Funct. Mater. 20, 3366–3372 (2010).

[b16] Zhang T. J. . Biotemplated synthesis of gold nanoparticle-bacteria cellulose nanofiber nanocomposites and their application in biosensing. Adv. Funct. Mater. 20, 1152–1160 (2010).

[b17] Zhu X. L., Feng C., Ye Z. H., Chen Y. Y. & Li G. X. Fabrication of magneto-controlled moveable architecture to develop reusable electrochemical biosensors. Sci. Rep. 4, 4169; doi: 10.1038/srep04169 (2014). 24566810 PMC3933910

[b18] Song J. . Synthesis of Au/graphene oxide composites for selective and sensitive electrochemical detection of ascorbic acid. Sci. Rep. 4, 7515, doi: 10.1038/srep07515 (2014). 25515430 PMC4268635

[b19] Rong Q. F., Han H. L., Feng F. & Ma Z. F. Network nanostructured polyprrole hudrogel/Au composites as enhanced electrochemical biosensing platform. Sci. Rep. 5, 11440, doi: 10.1038/srep11440 (2015). 26074185 PMC4466777

[b20] Lambert T. N. . Synthesis and characterization of titania-graphene nanocomposites. J. Mater. Chem. C 46, 19812–19823 (2009).

[b21] Li L. . One-step synthesis of polyhydroquinone-graphene hydrogel composites for high performance supercapacitors. J. Mater. Chem. A 3, 16033, doi: 10.1039/c5ta03881b (2013).

[b22] Wang Z. L., Guo R. Ding L. X., Tong Y. X. & Li G. R. Controllable template-assiated electrodeposition of single- and multi-walled nanotube arrays for electrochemical energy storge. Sci. Rep. 3, 1204, doi: 10.1038/srep01204 (2012). 23393615 PMC3566116

[b23] Liu N., Liu Z. M., Han H. L. & Ma Z. F. Graphene oxide reduced directly by redox probes for multiplexed detection of tumor markers. J. Mater. Chem. B 2, 3292–3298 (2014). 10.1039/c3tb21699c 32261591

[b24] Liu N. & Ma Z. F. Au-ionic liquid functionalized reduced graphene oxide immunosensing platform for simultaneous electrochemical detection of multiple analytes. Biosens. Bioelectron. 51, 184–190 (2014). 23962704 10.1016/j.bios.2013.07.051

[b25] Sabri Y. M. . Gold nanospikes based microsensor as a highly accurate mercury emission monitoring system. Sci. Rep. 4, 6741, doi: 10.1038/srep06741 (2014). PMC4206864 25338965

[b26] Xu S. J., Liu Y., Wang T. H. & Li J. H. Positive potential operation of a cathodic electrogenerated chemiluminescence immunosensor based on luminol and graphene for cancer biomarker detection. Anal. Chem. 83, 3817–3823 (2011). 21513282 10.1021/ac200237j

[b27] Xu T., Liu N., Ma Z. F. & Yuan J. Triple tumor markers assay based on carbon-gold nanocomposite. Biosens. Bioelectron. 70, 161–166 (2015). 25814405 10.1016/j.bios.2015.03.036

[b28] Zhu Q., Chai Y. Q., Yuan R. & Zhou Y. Simultaneous detection of four biomarkers with one sensing surface based on redox probe tagging strategy. Anal. Chim. Acta 800, 22–28 (2013). 24120163 10.1016/j.aca.2013.08.039

[b29] Zhu Q., Chai Y. Q., Zhuo Y. & Yuan R. Ultrasensitive simultaneous detection of four biomarkers based on hybridization chain reaction and biotin-streptavidin signal amplification strategy. Biosens. Bioelectron. 68, 42–48 (2015). 25562732 10.1016/j.bios.2014.12.023

[b30] Dong X. C. . 3D graphene foam as a monolithic and macroporous carbon electrode for electrochemical sensing. ACS Appl. Mater. Interfaces 4, 3129–3133 (2012). 22574906 10.1021/am300459m

[b31] Guo Y. J., Han Y. J., Shuang S. M. & Dong C. Rational synthesis of graphene-metal coordination polymer composite nanosheet as enhanced materials for electrochemical biosensing. J. Mater. Chem. 22, 13166–13173 (2012).

[b32] Sun X. B. & Ma Z. F. Highly stable electrochemical immunosensor for carcinembryonic antigen. Biosens. Bioelectron. 35, 470–474 (2012). 22444512 10.1016/j.bios.2012.02.061

[b33] Chang Y. Y., Xie S. B., Chai Y. Q., Yuan Y. L. & Yuan R., 3,4,9,10-Perylenetetracarboxylic acid/o-phenylenediamine nanomaterials as novel redox probes for electrochemical aptasensor systems based on an Fe_3_O_4_ magnetic bead as a nonenzymatic catalyst. Chem. Commun. 51, 7657–7660 (2015). 10.1039/c5cc00684h 25848657

