# Supplementary material for: Polyhydroquinone-graphene composite as new redox species for sensitive electrochemical detection of cytokeratins antigen 21-1
Source: Sci Rep. 2016 Jul 28;6:30623. doi: 10.1038/srep30623 (PMC4964632; doi:10.1038/srep30623)
Supplement: Supplementary Information [file srep30623-s1.doc]

Supplementary Information

**Poly****hydroquinone-graphene composite as** **new redox species for sensitive electrochemical detection of cytokeratins antigen 21-1**

Huiqiang Wang^1^, Qinfeng Rong^1^, Zhanfang Ma*

Department of Chemistry, Capital Normal University, Beijing 100048, China


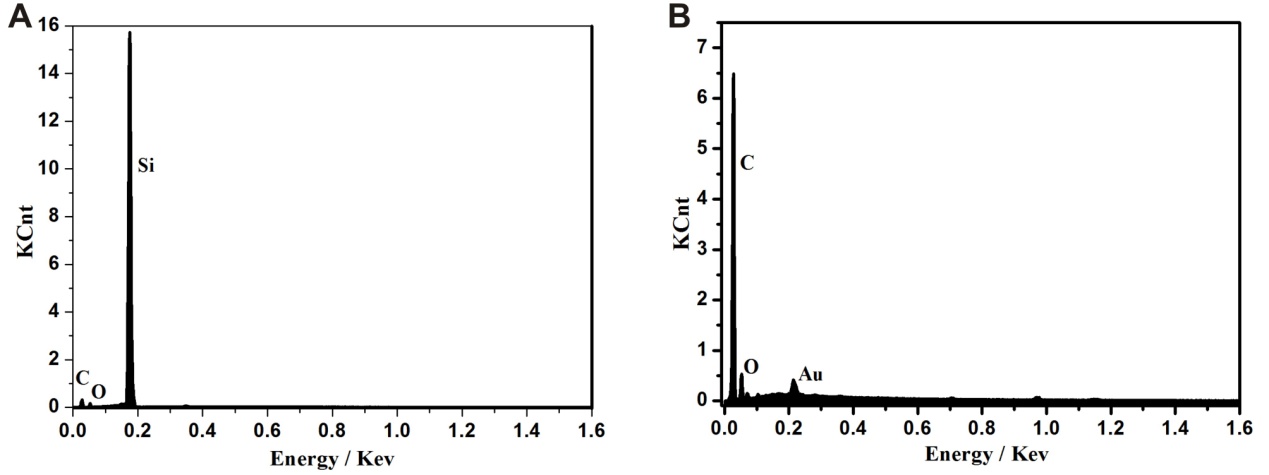


**Figure S1.** EDS of rGO&PHQ (A) and rGO&PHQ-Au (B).

**
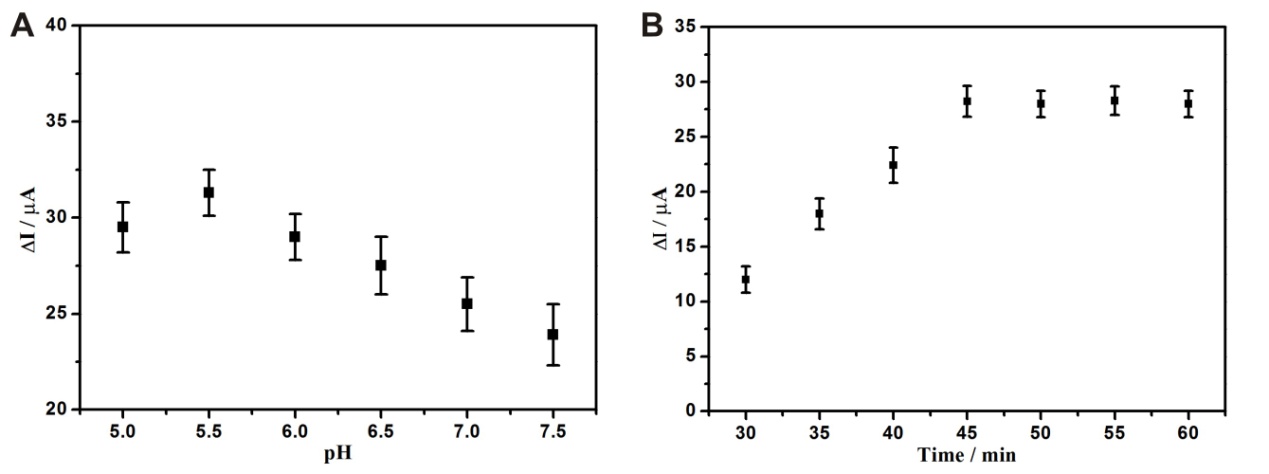
**

**Figure S2.** Effect of pH of electrolyte (**A**) and incubation time (**B**) on the DPV currents.


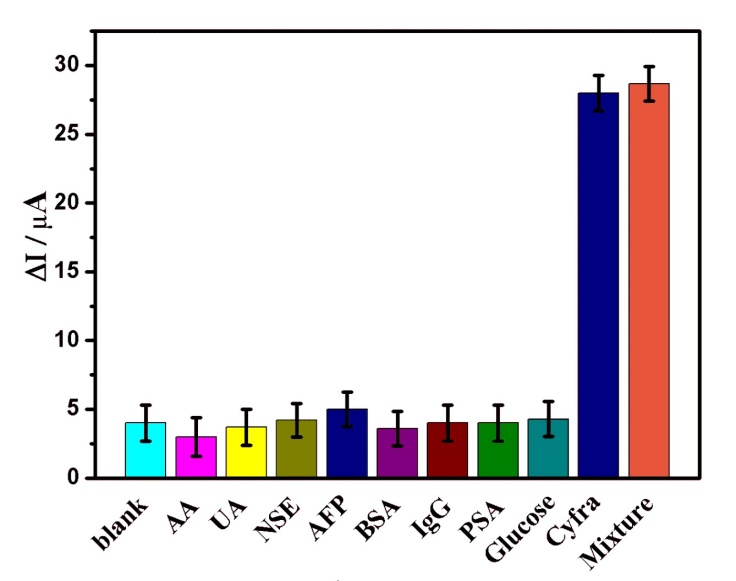


**Figure S3.** Anti-reference ability of the immunoassay (The error bars are standard deviations for n=3). The concentrations of AA, UA, NSE, AFP, BSA, IgG, PSA, and glucose were 100 ng mL^−1^. The mixture contains AA (100 ng mL^-1^), UA (100 ng mL^-1^), NSE (100 ng mL^-1^), AFP (100 ng mL^-1^), BSA (100 ng mL^-1^), IgG (100 ng mL^-1^), PSA (100 ng mL^-1^), glucose (100 ng mL^-1^) and CYFRA21-1 (5 ng mL^-1^).
